# Ten Simple Rules for Effective Online Outreach

**DOI:** 10.1371/journal.pcbi.1003906

**Published:** 2015-04-16

**Authors:** Holly M. Bik, Alistair D. M. Dove, Miriam C. Goldstein, Rebecca R. Helm, Rick MacPherson, Kim Martini, Alexandria Warneke, Craig McClain

**Affiliations:** 1 UC Davis Genome Center, University of California—Davis, Davis, California, United States of America; 2 School of Biosciences, University of Birmingham, Birmingham, United Kingdom; 3 Georgia Aquarium Research Center, Atlanta, Georgia, United States of America; 4 California Sea Grant, La Jolla, California, United States of America; 5 Department of Ecology and Evolutionary Biology, Brown University, Providence, Rhode Island, United States of America; 6 Pelagia Consulting, San Francisco, California, United States of America; 7 Joint Institute for the Study of Atmosphere and Ocean, University of Washington, Seattle, Washington, United States of America; 8 NOAA/Pacific Marine Environmental Laboratory, Seattle, Washington, United States of America; 9 Department of Biology, San Diego State University, San Diego, California, United States of America; 10 National Evolutionary Synthesis Center, Durham, North Carolina, United States of America; National Institutes of Health, United States of America

Online science outreach is paradoxically both easy and difficult. While anyone can start a blog and post updates to Twitter, it can be extremely challenging to establish a long-term following and demonstrate solid measures of success. A daunting number of online tools and platforms exist, and choosing where to start can be a difficult task in itself (for an explanation and guide to online tools, see [1]). As practicing scientists who have contributed to the highly visited marine science blog Deep-Sea News (DSN) for up to nine years, we provide guidance on how scientists, who often have minimal excess time and more pressing priorities, can maximally utilize new media tools. Here, we describe ten rules for conducting effective online outreach, so that other scientists can also enjoy the advantages of disseminating their knowledge and expertise through social media.

## Background and Overview of Deep-Sea News

Deep-Sea News (http://deepseanews.com) was established in 2005 with the sole purpose of tracking new literature for deep-sea scientists. Over the years, DSN content gradually transformed into a blog, and then incorporated other social media tools to become a multichannel online outreach platform. Currently, DSN is written by eight scientific professionals (the authors of this manuscript, with expertise in genomics, computational biology, ecology, oceanography, and conservation), with a collective expertise across a wide range of marine and oceanographic sciences. Capitalizing on our diverse interests and various social outlets, our readership has expanded to make DSN one of the most popular marine science blogs on the Internet, with ~7,000 average hits/day, and more than 8,000,000 cumulative hits. Because of our high visibility, DSN authors are routinely cited by various science journalists and quoted in prominent general audience publications such as Slate, Wired, National Geographic, and the annual print anthology The Open Laboratory: The Best Science Writing Online. We believe that the success of Deep-Sea News as an effective tool for online outreach is best illustrated by our mission statement: our commitment to “demystify and humanize science in an open conversation that instills passion, awe, and responsibility for the oceans” (http://deepseanews.com/about-2/mission/).

## Rule 1: Stop Treating Outreach and Research As Separate Entities

Integrating outreach tightly with ongoing research projects is key for maximizing success and minimizing time investment. Consider writing blog posts about either your newly published paper, literature you have compiled for manuscripts or grant writing, or literature you are reading for journal clubs. Blog posts focused on papers you have co-authored can serve as an important long-term resource, both as a first-person press release and a reference point for journalist enquiries. One example of research-outreach integration at DSN is the “Sizing Ocean Giants” project (http://www.storyofsize.com/sizing-ocean-giants/), an undergraduate research project focused on marine organism body size that included a heavy social media component. Undergraduates were asked to use social media to engage the public about their specific animals, leveraging online platforms as a pedagogical tool to increase their understanding of their organisms and the scientific process. Social media also served as a mechanism to engage other researchers. For example, a marine mammalogist reached out to the group via Twitter and ended up contributing large datasets to the project.

Since career advancement in the sciences depends on research productivity, it is important to link traditional metrics with online work. Based on our own experience, new grant proposals, research collaborations, and manuscripts (e.g., this article) can often result from online interactions. It should also be acknowledged that the requirement of translating research to a public audience increases both awareness and intimacy with the published literature—one that can feed directly back into your research program. Researchers can benefit from engaging with a broader set of disciplines than one would normally interact with, and these combined factors can lead to origination of new research ideas.

Online outreach projects can be designed and organized in a way that equates to publishable units; for example, can the data ultimately be used as the basis of a research paper, or can the outreach experience itself represent a useful case study that could be conveyed as an editorial or commentary? Research and teaching activities should also be considered in light of the outreach potential. In the Sizing Ocean Giants project described above, students used new media tools as part of their class projects. Another example is tweeting from conferences, which can quickly broaden your professional network. Considering the above points will ensure that your outreach activities and social media use work hand-in-hand with your research program. We also note here that building a track record in online outreach is, in itself, important for justifying your ability to successfully execute Broader Impacts sections of grant proposals.

## Rule 2: Be Strategic. Be Deliberate

Before embarking on any actual online outreach, the first step should be to define your overall short-term and long-term goals. We believe that it is important to incorporate some type of formal planning mechanism for all online outreach projects; planning is key for defining, measuring, and evaluating success over the course of the project. The DSN mission statement and core values (http://deepseanews.com/about-2/mission/) resulted from a facilitated, in-person blog retreat in October 2011. DSN needed a clear vision as to where our social media outreach was headed, including our niche, our goals, and our values. We could not measure success unless we defined what success was. Furthermore, it was important to explicitly iterate these ideas on the website itself, so that our audience knew what to expect from us.

Despite the informality of our group, we took this process very seriously and ran the retreat as a mediated strategic planning session, complete with mission statements, value propositions and action items. This clarifying of our mission and core values allowed us to build more effective strategies for generating content, attracting new members, and building an audience. As a result, our website growth in 2012 (as measured by daily hit rate) changed from a linear to an exponential growth trajectory, which continues until today.

## Rule 3: Find *Your* Niche and Story

After drafting your outreach goals, the next step is to determine your target audience, define your outreach angle (i.e., a writing voice or online persona), and determine the online tools that are best suited to your needs. Don’t assume that online audiences are only interested in dinosaurs, sex, or chocolate (reoccurring subjects on many mainstream science blogs)—let your own scientific passions drive the content you generate and curate (see also Rule 6). For example, Chris Mah, a researcher at the Smithsonian Institution, is unapologetic about his enthusiasm for echinoderms (http://echinoblog.blogspot.com/). Also keep in mind that any posts that go viral (or touch on controversial topics) can swing a reader’s view of what a blog is all about. Because it is difficult to predict popularity, it is worthwhile to ask if a post’s content is representative of how you want people to know your blog.

Here we note that outreach projects should consciously choose between “inreach” versus “outreach.” Inreach refers to discussion and sharing amongst a known, closed group (most likely a community to which you already belong). Using Twitter to discuss a research technique with colleagues in your subdiscipline is one example of inreach. Other examples may include submitting code patches on GitHub, or commenting on peer-reviewed publications via PubMed commons. Although it may increase the visibility of the conversation due to the public nature of the platform, the discussion is not likely to extend beyond scientists, and certainly not to the general public. Outreach, on the other hand, refers to a dedicated and sustained effort to disseminate science beyond the ivory tower, for example, working with journalists to discuss new research in the mainstream media, or conducting an “ask me anything” live blogging session on a site such as Reddit. Your specific goals will determine whether your project is best suited to inreach, outreach, or some combination of the two. There is a place for both avenues online, but remember that these two concepts are not the same at all.

## Rule 4: Branding…Branding…Branding…

Branding may seem “corporate”, but style and consistency are both critical for online outreach. Branding is a powerful tool. In the corporate arena, a well-established brand can be the key to a successful business enterprise. The same ideology holds true in the online ecosystem. Fundamentally, a brand represents a promise to your audience that you will abide by a certain set of principles or a mission statement. The best brands are ones that instill and inspire others to extend a personal interest and trust in the message the brand exemplifies. This brand should permeate all aspects of your platform and instill a sense of quality, credibility, and experience. However, it is important to note that such a trust does not happen quickly, and effective branding can require a significant investment of time and energy.

Before officially branding any online outreach platform, you must first solidify the message you are trying to convey and the audience for whom it is intended. At DSN, our mission statement and set of core values has set forth a clear and consistent style—a style which our readership has come to expect. This standard allows a diversity of voices to be present through multiple outlets, while keeping those voices true to our common objective. We believe our emphases on “saying things that others do not” and “awareness through scrutiny, not negativity” (http://deepseanews.com/about-2/mission/) have greatly added to our brand and successes in online outreach. Moreover, we have found that having a clean, easily accessible, and visually appealing interface is also beneficial for branding and building readership. Most importantly, we have chosen a distinctive symbol, the giant squid, that is consistent, recognizable, and personifies what DSN stands for (http://deepseanews.com/2011/06/from-the-editors-desk-the-giant-squid-can-be-a-panda-for-the-ocean/). Our logo also incorporates a homage to marine field work (red/white diagonal akin to a SCUBA flag), and a sense of playfulness (a pirate’s eyepatch). Taken together, our branding encapsulates the underlying core values and mission of DSN.

## Rule 5: Recruit a Top-Notch Team

Because social media is so much work, distributing effort and delegating tasks amongst different participants can greatly increase long-term sustainability. Online outreach requires producing regular content, appealing to diverse users, and building a long-term community of readers and commenters. A frequent supply of unique content is critical for building and sustaining an online following [2], and also represents one of the most challenging aspects of maintaining a single author blog. Group blogs are thus one of the best ways to minimize time investment and help maximize outreach efficiency: a group blog has the potential for more diversity (DSN includes female and LBGT voices) and reduces the burden on each individual. Group blogs can also invite posts from guest contributors, giving exposure to new scientists and helping to further broaden blog content. If you are interested in blogging, don’t be afraid to ask an established blog about submitting a guest post—many sites welcome such contributions. A mixture of regular and guest contributors will naturally help to disseminate content more widely, since group blogs inherently leverage each person’s own personal and professional networks. Regardless of the makeup of your blogging team, it is still vital to focus on publishing credible science using good communication skills.

## Rule 6: Focus on the Story

Producing something popular on the Internet is as much about passion and storytelling as it is about good content. The best content in the world, if delivered in a drab or ineffective manner, will not reach its desired audience or will fail to engage their attention. With passion, the right writing style, and deft use of digital media tools, you can make any type of science cool, and the importance of making things cool cannot be overstated.

Dickson [3] coined the term Information Deficit Model (also known as Science Deficit Model or just Deficit Model) for the notion that public mistrust of science results from a lack of understanding of scientific topics. The logical corollary of the IDM is that if we can simply overcome the knowledge deficit, public trust in science will improve. In our experience, there is no evidence that this actually happens. If we simply put the knowledge out there, most people will still lack the conceptual context to understand and acknowledge the significance of what they are being shown. We must not simply communicate the content of science, but also a passion for science. It is passion that is contagious and passion that drives scientists to push the frontiers of knowledge and understanding of the natural world. It will be passion that elevates the public to a greater appreciation of the transformative power of science; the knowledge deficit will take care of itself. Consider the success of the television series *Cosmos*, *The Undersea World of Jacques Cousteau*, and the BBC documentary works of Sir David Attenborough. In all three cases, there is incredible scientific content, to be sure, but the true success of these programs lies in their presenters—charismatic and authentic scientist/explorers who share their passion first and foremost, and the scientific content rides along with it.

One way that scientists can help convey passion is through storytelling. The notion of narrative structure is familiar to everyone, often unconsciously so, which provides great potential for scientific material that can be delivered in this fashion. Use of storytelling mechanisms employed by writers and journalists can help tremendously, as can explicit collaboration with artists, filmmakers, and other narrative experts. For writers, books such as “A Field Guide for Science Writers” [4] are invaluable resources for conducting effective outreach. In terms of visual media, there are many excellent outreach initiatives emerging on YouTube, such as “Minute Physics” (https://www.youtube.com/user/minutephysics), and “The Brain Scoop” produced by the Chicago Field Museum (https://www.youtube.com/user/thebrainscoop). RadioLab (http://www.radiolab.org/) is yet another enthralling example of scientific ideas conveyed in audio form, via podcast and public radio.

Another approach is to bridge the cultural gap between scientists and the public by explicitly creating commonalities with the reader. Scientists are not separate from the rest of society; we are also members of the public. We shop for groceries, visit the dry cleaners, take our kids to school, and vote in elections. We are influenced by society and engage in popular culture, and thus our communications and narratives can be deeply rooted in this idea. At DSN we aim to integrate pop culture with scientific content. This leverages virality and the vast exposure of pop culture phenomena, but also serves to show that the authors themselves are not aloof ivory tower-dwellers. For example, recent DSN posts have referenced Miley Cyrus (http://deepseanews.com/2014/02/mooring-family-photos/) and incorporated Internet memes (http://deepseanews.com/2013/09/lol-ocean-giants/).

## Rule 7: Leverage Multiple Tools to Disseminate Content and Build Up Your Network

We strongly encourage the use of multiple online tools, in order to reach different audiences and drive traffic to the main blog or website. Most readers tend to use one or two new media tools, with platform use depending on users’ personal preferences and established online social networks. Thus, it is important to cross-promote new content to Tumblr, Twitter, Facebook, Pinterest, and other relevant platforms. At DSN, we use a division of labor for these types of cross-promotion, relying on authors that specialize in each different tool. We also try to automate as much of this process as possible, which is imperative for time management and efficiency in outreach activities. A suite of Wordpress plugins and standalone websites can be leveraged for automatically pushing content to different social media accounts (some examples include http://dlvr.it and http://twitterfeed.com), although such automation is best supplemented and balanced with human-led interaction with online audiences.

Actively engaging with audiences, not just broadcasting information, is also an important part of using these different tools. Kietzmann et al. [5] cite examples from the corporate world, where ineffective social media engagement (ignorance or misguided policies), can result in both missed opportunities and failure to mitigate potential bad press. For scientific outreach projects, monitoring accounts and participating in subsequent discussions (e.g., responding to Facebook/Blog comments, answering questions on Twitter) is a key component for sustaining participation from followers and encouraging growth of your online community. Social media engagement therefore helps audiences pursue their own interests, helps scientists address controversial topics like climate change, and simultaneously helps break down perceived barriers between scientists and society (see Rule 6).

Finally, we note that open licensing of online content can play a pivotal role in its dissemination. All content at Deep-Sea News is available under a Creative Commons (CC) license (http://creativecommons.org/), allowing users to freely share and redistribute the material with attribution to the original source. CC-licensed materials can be a long-term boon to outreach efforts, especially if content is widely shared and linked on sites such as Wikipedia. For example, the photo-sharing website Flickr, http://www.flickr.com, allows users to post and search for CC-licensed images. Data and figures can also be posted to repositories such as Figshare (http://figshare.com) where they are assigned a unique digital object identifier (DOI), making them both shareable and citable. Open content thus allows your outreach project to be built on and complemented by other people, by providing unique materials for audiences to engage with and share.

## Rule 8: Collect and Assess Data

Currently, there is much anecdotal and observational evidence regarding what scientists gain through the use of new media tools (in terms of professional benefits and outreach impacts), but not much in the way of systematic data and results. At Deep-Sea News, we attempt to collect as much data as feasible in order to gauge traffic spikes, content-related trends, and long-term growth in readership. Website metrics such as Google Analytics (http://www.google.com/analytics/) and Stat Counter (http://statcounter.com) are used to track site traffic, including the point of entry (page and/or search engine term), country of origin of visitors, and unique versus repeat visitors. Blog posts are specifically tailored with Search Engine Optimization plugins installed via Wordpress—each post has a category, keyword tags, and a descriptive blog title post that helps to drive search engine traffic. In addition, we keep track of social media metrics: number of tweets/retweets, Facebook “likes,” and other shares of a given post.

Other strategies and metrics we use at DSN include blog comments and reader surveys. Reader comments are also an important measure of impact. Some blog posts inspire further conversation in the comments section that can extend to other social media platforms such as Twitter or Facebook. However, oftentimes the quantity of blog comments correlates to controversy more than popularity, and can be counterproductive to our science education mission. Therefore, to keep the comment section restricted to productive discussion, DSN has implemented clear moderation policies (http://deepseanews.com/about-2/commenting-policy/).

Despite this suite of metrics, as blog administrators we are often uncertain about how to best interpret and maximize the use of analytic data. For example, posts published at DSN for 2013 (*n* = 299), garnered a total of 1,666,119 page views. Of these views, 82.6% were received on the top 20 posts; the lowest ranking 200 posts accounted for just 5% of total 2013 views. If anything, these trends illustrate the asymmetry of online reach and impact. Many posts receive a moderate amount of interest and reader comments, but occasional posts go viral and attract mainstream media attention, which has a dramatic positive effect on blog traffic. It is difficult to predetermine what posts will soar in popularity, but the longer you participate in effective online outreach, the more it is likely to happen. In this sense, reaching a truly large audience is a long game.

## Rule 9: Iteratively Assess What Works and What Doesn’t

The above-mentioned analytics can be used to assess your online reach and track progress towards your goals. However, the online environment is still an evolving and untested sphere, and you will undoubtedly have to adopt a trial-and-error approach to find what works best. Reflecting on the 3,688 posts currently hosted at Deep-Sea News, we can provide some insight into the kinds of posts that work versus those that do not. Post length is an important aspect to consider, since different lengths can target different audiences and outreach goals. In general, posts that are well received on our site are usually 400–800 words, with liberal use of images and videos. Lists are particularly low-hanging fruit and often go viral (e.g., “Top Ten. . .” or “Best of..” posts). Occasional long posts (>800 words) can cater towards a more engaged, but smaller audience. Since these posts require more of the reader’s time, they tend to be popular amongst people with an existing interest in science, or students and teachers seeking educational resources. Longer posts can also be quite effective in addressing public misconceptions, which often take considerable time to untangle.

Tone is a critical part of outreach identity. Successful writers often have a distinctive voice, which creates an interesting and engaging narrative. However, many writers must find their voice, rather than knowing it from the onset. The web is a fantastic place to try new writing styles, especially with near instant feedback in the form of Facebook likes and shares, tweets, or recognition from fellow bloggers. At DSN, we have found that humor is key. Humor, when used effectively, generates a relaxing and welcoming environment for the reader. Linking to pop culture or Internet memes also connects posts to a larger social context and can help people relate their lives to the science being discussed.

In the course of DSN’s history, we have also realized that there are real barriers to good science outreach. First, crafting a good post is time-consuming, and can take many hours. By spreading the work around, group blogs help to alleviate some of the time pressure (Rule 5). Certain topics are also inherently more difficult to tackle, such as Fieldwork or Expedition blogging. In order to do this type of writing well, a strong “hook” and/or human interest is needed to draw the reader in—otherwise these types of posts are akin to sitting through a slideshow of someone else’s travel photos. Controversial topics can also be difficult to address, as they often draw unwanted attention, criticism, and negativity to the blog—examples at DSN include fisheries, climate change, and the Sea Shepherds organization.

## Rule 10: Create Prestige for Public Scholarship

What do we truly gain from online outreach activities? As scientific professionals working in the research, academia, and nonprofit sectors, we are not evaluated on our outreach. However, we argue that there are a number of personal and professional benefits to be gained, as discussed in previous publications [1,6]. The most important overarching benefit is visibility—to one’s colleagues, to the media, and to the public. By being accessible, researchers participating in online conversations have the opportunity to have a much more influential voice for their science. In these days of dwindling governmental investment and increased public distrust of science, scientists need to speak out on the value of their profession and training.

At DSN, we have derived professional benefits and personal satisfaction from our work, including published papers [1], new collaborations (e.g., http://deepseanews.com/2012/08/sharks-and-lasers-not-just-for-entertainment/), and substantial media coverage of our work (e.g., http://articles.latimes.com/2014/jan/12/local/la-me-west-coast-radiation-20140113). However, the most rewarding aspect is to have become an authoritative source on ocean science for the media and the public. Some of our writing, such as that on the Fukushima nuclear disaster and the Deepwater Horizon oil spill, has been widely quoted in the press, and our posts aimed at students interested in marine science are used by teachers and advisors around the world. Because we have witnessed such direct and beneficial gains as a result of our online outreach activities, we feel strongly that such activities should be given more weight when determining scientific productivity, e.g., during hiring/promotion decisions. The impact of online activities is increasingly recognized [7–8], and they should be formally encouraged.

## Conclusions

In the end, it’s important to have reasonable expectations for your online activities. Don’t be afraid to start small. Remember that not every single one of your posts will go viral—in fact, it will be very rare that they do. Online outreach is generally a long game. Content production and consistency are key factors that will impact how audiences view and access your blog. Finally, quality and engagement are both important for becoming a trusted go-to source in the online world, and for extending your impact beyond the Internet.
